# Application of Anterior Segment Optical Coherence Tomography in Pediatric Ophthalmology

**DOI:** 10.1155/2012/313120

**Published:** 2012-08-09

**Authors:** Ricardo Salles Cauduro, Caroline do Amaral Ferraz, Maira Saad Ávila Morales, Patricia Novita Garcia, Yara Cristina Lopes, Paulo Henrique Souza, Norma Allemann

**Affiliations:** ^1^Department of Ophthalmology, Federal University of São Paulo (UNIFESP), 04023-062 São Paulo, Brazil; ^2^Fleury Medicina e Saúde, Ophthalmology Diagnostic Center, 04501-900 São Paulo, Brazil; ^3^Department of Ophthalmology, Santa Casa de Misericórdia de São Paulo, 01221-020 São Paulo, Brazil; ^4^Department of Ophthalmology & Visual Sciences, University of Illinois at Chicago, Chicago 60612, USA

## Abstract

*Purpose.* Application of anterior segment optical coherence (AS-OCT) in pediatric ophthalmology. *Methods.* Retrospective clinical study case series of 26 eyes of 19 pediatric patients throughout a 21-month period, presenting anterior segment pathologies, were submitted to AS-OCT examination (OCT Visante, 1310 nm, Zeiss), noncontact technique, no sedation requirement. *Results.* AS-OCT images were obtained from 19 patients (range: 2 months to 12 years). Clinical diagnosis of anterior segment abnormalities included cornea disease (*n* = 7), congenital anterior segment conditions (*n* = 10), ocular trauma (*n* = 1), anterior segment surgeries (*n* = 2), iridocorneal angle abnormalities (*n* = 4), intermediate uveitis (*n* = 2). The most common OCT findings were corneal hyperreflectivity and thickening (*n* = 15), shallow anterior chamber with iris-lens diaphragm anterior displacement (*n* = 4), atypical corneal curvature (*n* = 4), corneal thinning (*n* = 4), peripheral synechiae with angle closure (*n* = 3), increased anterior chamber depth (*n* = 2), and proximal portion of glaucoma drainage tube (*n* = 2). *Conclusion.* In the present study, noncontact AS-OCT demonstrated to be a feasible technique to evaluate the anterior segment providing anatomic details and useful to clarify diagnosis in the pediatric population.

## 1. Introduction

Optical coherence tomography (OCT) is a high-resolution imaging technique, which allows a noninvasive tissue observation through sectional cuts of the ocular structure. This concept is based on the measurement of low-coherence delay infrared light reflected onto a tissue to be examined with the use of interferometry [1, 2]. The system with a wavelength of 1310 nm has allowed greater penetration into opaque tissues such as sclera and limbus for the visualization of angular structures [1–4]. Anterior segment OCT (AS-OCT) technology has improved in the last years with the development of higher resolution systems, considering time domain and Fourier-domain systems.

In most cases, OCT has its usage limited to the adult population. The role of anterior segment OCT in ocular pediatric diseases has not been well reported [5]. Anterior segment anatomy evaluation in children is challenging and was restricted to high-frequency ultrasound techniques (UBM), which required immersion technique and sedation [6].

## 2. Patients and Methods

Retrospective analysis of patients under 12 years of age presenting with anterior segment conditions and requiring imaging techniques for elucidation were submitted to anterior segment OCT examination (Visante OCT, 1310 nm, Carl Zeiss Meditec Inc., Dublin, USA), during a 21-month period (January 2008 to September 2009): protocol approved by the UNIFESP Ethics Committee in Research under the number CEP/UNIFESP no. 0788/09.

Presumed ocular diagnosis was considered in comparison to AS-OCT findings.

For AS-OCT examination no sedation was necessary; anesthetic eye drops or eyelid speculum was not routinely used, except for one only eye of a 2-month-old patient who required the use of topical hydrochloride proxymetacaine 0.1% (Alcon). All images were acquired in standard light conditions using the anterior segment single, dual, and/or quad scan (scan width: 16 mm; scan depth: 8 mm) and cornea single and enhanced cornea protocols (scan width: 10 mm; scan depth: 4 mm) by two examiners (PHS, NA), adding the pachymetric map, when needed. Poor vision, lack in fixation or nystagmus usually determine poor perpendicularity. Images were scanned at the horizontal meridian (0° and 180°) and, when possible, also at the vertical meridian (90° and 270°) and the oblique meridians (135° and 315°, 45° and 225°).

The cross-sectional AS-OCT images with the best quality were further analyzed using software provided by the manufacturer. This included the measurement of the following parameters: cornea thickness, anterior chamber depth (ACD), internal AC diameter, evaluation of the anterior chamber angle (ACA). Qualitative analysis was used to evaluate corneal and lens reflectivity, to describe synechiae and malformations, and to analyze internal structure of superficial lesions.

## 3. Results 

The sample included 19 patients, their age ranging from 2 months to 12 years old.

Anterior segment OCT exam was performed in 26 eyes with presumed diagnosis of anterior segment.

Demographic data and presumed diagnosis were listed in Table 1. AS-OCT findings related to diseases of the anterior segment were grouped in Table 2.

Congenital anterior segment changes were the most frequent indications including mucopolysaccharidosis (*n* = 4), keratoconus (*n* = 3), and microphthalmia (*n* = 3).

Corneal changes such as increased reflectivity (*n* = 9) and thickening (*n* = 6) were the most observed tomographic findings, as referred to in Table 3.

### 3.1. Application of AS-OCT in Cornea-Related Diseases

**Figure 1:**
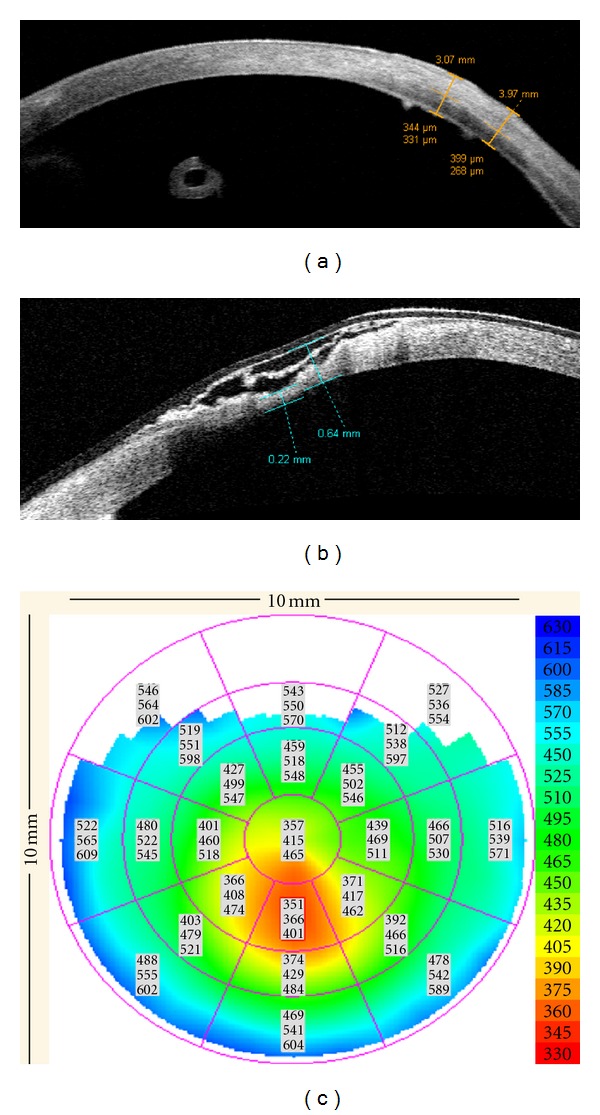
Anterior segment OCT images of corneal changes in the pediatric population. (a) Increased stromal reflectivity (opacity) measured at 399 microns depth, residual stroma = 268 microns. Note a circular structure in the anterior chamber correspondent to a transverse section of a glaucoma drainage tube. (b) Self-sealing perforating corneal injury treated with adhesive (hyper-reflective image at the surface of the cornea with a bandage contact lens), underlying stromal thickness = 220 microns. (c) Pachymetric map in a keratoconus patient, thinnest area inferiorly colored in red (total corneal thickness inferiorly = 352 microns).

Preoperative evaluation of corneal opacities, providing accurate measurement of the scar depth (range: 344 to 399 microns) and of the residual stroma thickness, *n* = 15 eyes; and postoperative evaluation of corneal penetrating transplant (donor recipient wound evaluation and pachymetric map, *n* = 1) and glaucoma drainage tube positioning in relation to the peripheral cornea (*n* = 2) as in Figures 1(a) and 3(b). In case of graft failure an overall thickening of the cornea would be detected (Figure 3(a)). One case (Case 8, Table 3) presented a self-sealing perforating corneal injury and was handled with bandage contact lens and local adhesive application; AS-OCT allowed to evaluate the underlying residual stroma thickness (220 microns, Figure 1(b)). In one eye of an 11-year-old child with keratoconus, AS-OCT provided the pachymetric map objectively measuring and localizing corneal thinning (352 microns, Figure 1(c)).

### 3.2. Application of AS-OCT in Developmental Anomalies of the Anterior Segment

**Figure 2:**
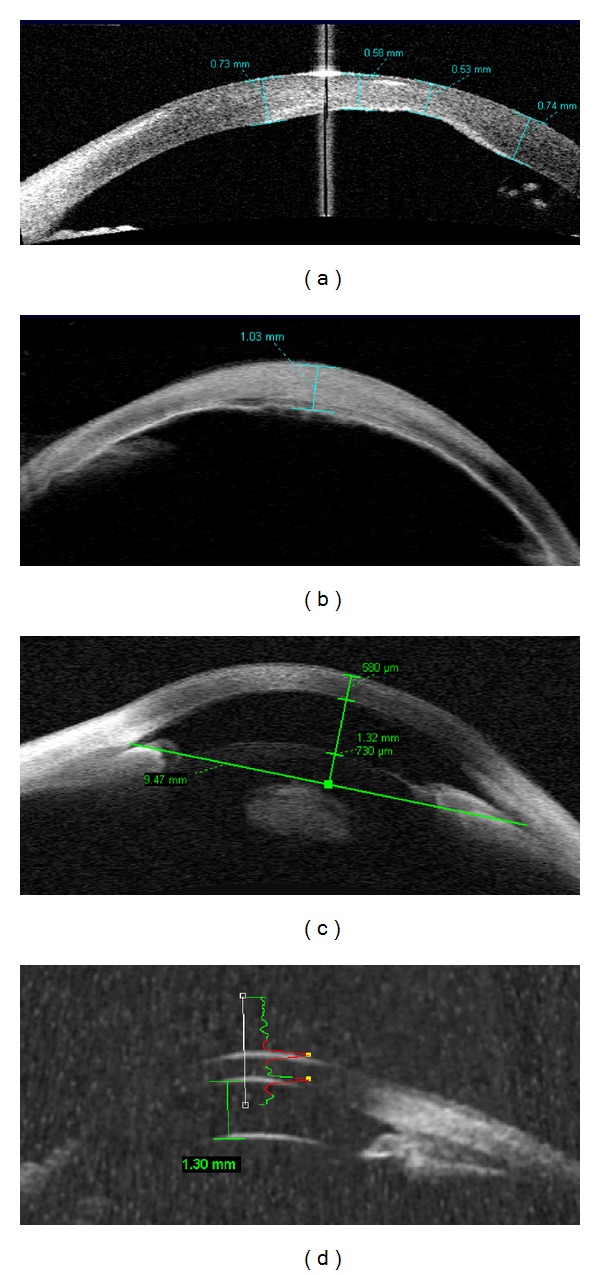
Anterior segment OCT images of developmental anomalies in a pediatric population. (a) Meso-ectodermal dysgenesis, Peters anomaly: localized stromal hyper-reflectivity, posterior corneal defect, increased corneal thickness (580 microns at the hyper-reflective area, 740 microns in adjacent area). (b) Corneal thickening and hyper-reflectivity = 1,003 microns. (c) Anterior chamber depth (1.32 mm) and inner AC diameter (9.47 mm) presented under normal values, compatible with microphthalmia. At axis 180, iris demonstrated a short length. (d) UBM of the same patient at axis 90, confirmed ACD = 1.30 mm.

**Figure 3:**
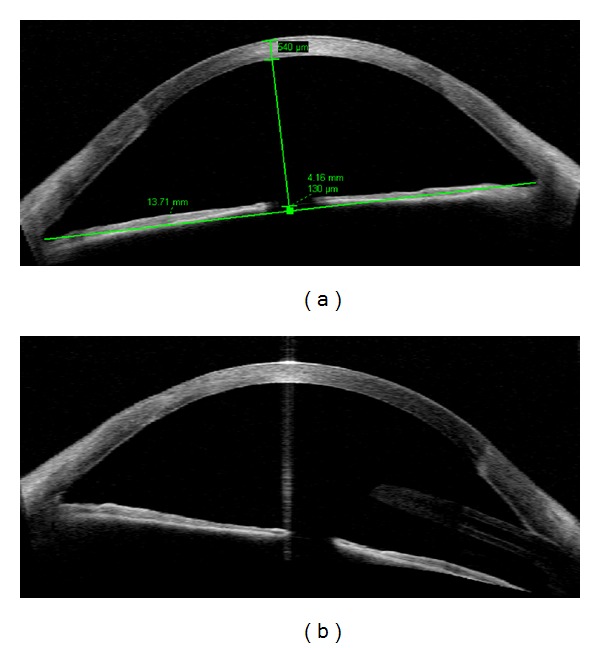
Postoperative evaluation of the pediatric population using AS-OCT. (a) Penetrating corneal transplant, donor-recipient junction evaluation demonstrates no gap or step. (b) Donor-recipient junction and an inferonasal glaucoma drainage tube, apparently touching the cornea, that presents local edema (right side).

Mesoectodermal dysgenesis (as Peters syndrome, 2 eyes) demonstrated increased reflectivity of the central cornea (leukoma) associated with local indentation (malformation) of the posterior surface (Descemet's membrane defect, Figure 2(a)), with overall increased stromal reflectivity and thickness (Figure 2(b)). Findings of decreased anterior chamber depth and peripheral synechiae were associated (*n* = 2 eyes).

### 3.3. Applications of AS-OCT in Microphthalmia

Reduced anterior chamber depth (*n* = 1 eyes, ACD = 1.32 mm) and inner AC diameter (9.47 mm). Both eyes were also submitted to high frequency ultrasound with a 50 MHz transducer, and measurements were comparable (Figures 2(c) and 2(d)).

### 3.4. Application of AS-OCT in Evaluating the AC Angle

**Figure 4:**
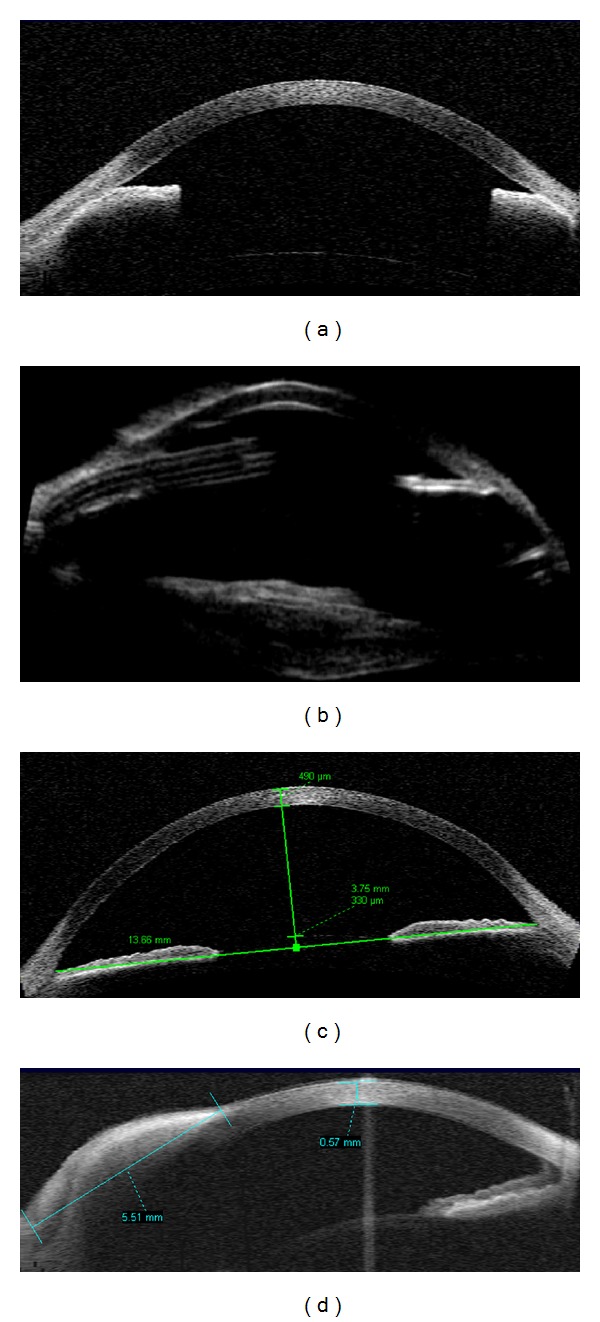
(a) Anterior segment OCT in pediatric patients. Neovascular glaucoma and secondary angle closure (anterior synechiae). (b) High frequency ultrasound (UBM) of the same eye after implantation of glaucoma drainage tube, showing the tube in the anterior chamber, an irregular and hyperrefletive lens and angle closure at the opposite position (c) AS-OCT in a congenital glaucoma with anterior chamber depth = 3.75 mm. (d) Corneo-scleral dermoid cyst with posterior attenuation, radial extension = 5.51 mm.

Two eyes with neovascular glaucoma and vitreous hemorrhage presented generalized angle closure. UBM was performed after glaucoma drainage implantation (Figures 4(a) and 4(b)). Congenital glaucoma (*n* = 2 eyes) presented wide angle, increased AC depth (3.75 mm), and inner AC diameter (13.66 mm), as in Figure 4(c).

### 3.5. Application of AS-OCT in Anterior Segment Tumors

Limbal dermoid cyst (*n* = 1 eye) presented as an elevated highly reflective lesion at the limbal region, that apparently occupied deep corneal stroma. Lateral boundaries were determined (radial measurement = 5.52 mm, Figure 4(d)), but due to local shadowing artifact, the posterior limit was uncertain.

## 4. Discussion

Anterior segment OCT is an ancillary exam suitable for glaucoma evaluation [1, 4], allows the measurement of the anterior chamber inner diameter [1, 3], and provides a pachymetric map [7], also suitable for evaluation of ectatic disorders [1], corneoscleral abnormalities [1], corneal transplant followup [1], and anterior segment tumors [1].

There are limitations in the evaluation through AS-OCT considering the lack of information of the posterior chamber, such as the ciliary body and sulcus. High-frequency ultrasound methods (35 to 50 MHz) usually allow better penetration into opaque and high-density structures [3], requiring an immersion technique and contact to the globe.

The usage of AS-OCT has been limited to the adult population [2, 4, 7]. The application of this method in the evaluation of pediatric ocular diseases has not been well defined [5].

In the pediatric population, reports using a posterior segment OCT system (820 nm) demonstrated mean values to the foveal thickness (186 microns) and nervous fiber layer thickness (108.27 microns), additionally allowing an analysis of the optical disc [8, 9] and a detailed morphological description of the macula in premature children [10]. The observation of prevalent intraocular tumors and simulatory lesions in children was also validated with posterior segment OCT method [5, 11]. Handheld posterior segment OCT devices are available to help surgeons at exams under anesthesia in the pediatric population.

Some eyes in the sample were evaluated with AS-OCT and comparatively with high frequency ultrasound (UBM) and similarities were reported.

Anterior segment OCT may be performed without difficulties in many children and its application will certainly expand relating to its non-contact and noninvasive scanning method. However, in some cases of non-cooperative children and children who present low visual acuity, condition which in other methods may not be relevant, the examination under anesthesia is recommended if a handheld AS-OCT device would be available [12, 13].

In the present study, AS-OCT was validated as a non-contact imaging method to evaluate the anterior segment of the pediatric population. In children, AS-OCT acquisition is considered more difficult because of the lack of cooperation in fixating. A concern was also the presence of dense corneal opacities, which could impede optimal imaging of the anterior segment structures. Both concerns did not prevent the acquisition of good quality AS-OCT images.

AS-OCT can be considered as a feasible imaging technique to be used with children and useful to clarify diagnosis and for clinical and/or surgical follow-up of anterior segment disorders. The development of handheld devices for anterior segment OCT evaluation is expected to improve the standard of medical care.

## Figures and Tables

**Table 1:** Characteristics of the pediatric population submitted to anterior segment OCT.

Patient no.	Gender	Age (years)	Eye	Presumed clinical diagnosis
1	M	6	OU	Mucopolysaccharidosis type IV
2	M	11	OS	Keratoconus
3	F	11	OU	Mucopolysaccharidosis type IV
4	M	2	OD	Dermoid cyst
5	F	12	OS	Perforating corneal trauma
6	M	0.17 (2 mo)	OS	Mesoectodermal dysgenesis
7	F	6	OS	Mesoectodermal dysgenesis
8	M	7	OU	Microphthalmia
9	F	11	OD	Cornea transplant and glaucoma drainage tube
10	F	5	OS	Sclerocornea
11	F	10	OD	Uveitis, peripheral anterior synechia and cataract
12	M	7	OU	Congenital glaucoma
13	F	11	OU	Intermediate uveitis
14	F	11	OU	Neovascular glaucoma
15	M	12	OU	Keratoglobus OD, keratoconus OS
16	F	12	OD	Glaucoma drainage tube
17	M	11	OS	Ciliary body lesion with anterior peripheral synechiae
18	F	5	OU	Congenital cataract, microphthalmia and iris coloboma
19	M	11	OD	Keratoconus and hydrops

Legend: M: male; F: female; OU: both eyes; OD: right eye; OS: left eye.

**Table 2:** Anterior segment pathologies in the pediatric population submitted to anterior segment OCT (AS-OCT) examination.

Anterior segment pathology	Number of eyes
Cornea diseases	
Mucopolysaccharidosis	4
Keratoconus	3
Anterior segment congenital condition	
Mesoectodermal dysgenesis	2
Sclerocornea	1
Congenital glaucoma	2
Microphthalmia	3
Keratoglobus	1
Ocular trauma	
Self-sealing corneal penetrating injury	1
Anterior segment surgery	
Cornea transplant and glaucoma drainage tube	1
Glaucoma drainage tube	1
Iridocorneal angle abnormalities	
Anterior synechiae post-uveitis	1
Neovascular glaucoma	2
Synechia associated to ciliary body lesion	1
Intermediate Uveitis	2
Ocular surface lesions	
Corneoscleral dermoid	1

**Table 3:** Anatomy-related classification of AS-OCT findings in a pediatric population with anterior segment pathologies.

Anatomic landmark	AS-OCT finding	Number of eyes
Cornea	Increased stromal reflectivity	9
	Thinning	3
	Thickening	6
	Stromal heterogeneity and irregular thickness	1
	Increased anterior curvature	3
	Increased posterior curvature	1
	Host-donor junction in corneal transplantation	1

Anterior chamber	Decreased anterior chamber depth	4
	Increased anterior chamber depth	2
	Position of glaucoma drainage tube	2

Crystalline lens	Congenital cataract	2

Iris	Peripheral synechiae	3

Sclera	Corneoscleral dermoid	1
